# Modality attention fusion model with hybrid multi-head self-attention for video understanding

**DOI:** 10.1371/journal.pone.0275156

**Published:** 2022-10-06

**Authors:** Xuqiang Zhuang, Fang’ai Liu, Jian Hou, Jianhua Hao, Xiaohong Cai

**Affiliations:** 1 School of Information Science & Engineering, Shandong Normal University, Jinan, China; 2 College of Intelligence and Information Engineering, Shandong University of Traditional Chinese Medicine, Jinan, China; Indian Institute of Technology Patna, INDIA

## Abstract

Video question answering (Video-QA) is a subject undergoing intense study in Artificial Intelligence, which is one of the tasks which can evaluate such AI abilities. In this paper, we propose a Modality Attention Fusion framework with Hybrid Multi-head Self-attention (MAF-HMS). MAF-HMS focuses on the task of answering multiple-choice questions regarding a video-subtitle-QA representation by fusion of attention and self-attention between each modality. We use BERT to extract text features, and use Faster R-CNN to ex-tract visual features to provide a useful input representation for our model to answer questions. In addition, we have constructed a Modality Attention Fusion (MAF) framework for the attention fusion matrix from different modalities (video, subtitles, QA), and use a Hybrid Multi-headed Self-attention (HMS) to further determine the correct answer. Experiments on three separate scene datasets show our overall model outperforms the baseline methods by a large margin. Finally, we conducted extensive ablation studies to verify the various components of the network and demonstrate the effectiveness and advantages of our method over existing methods through question type and required modality experimental results.

## Introduction

In recent years, studies on video question answering (Video-QA) based on both vision and natural language have successfully benefited from deep neural networks [1–5]. This task aims to select the reasoning process of the correct answer from the answer candidates in the video [6]. Machine’s under-standing of images and videos is transitional from labeling the image with a few words to learn to generate a complete sentence.

Most existing methods [7–11] attracted a lot of attention and Video-QA has experienced outstanding advancement. Weinzaepfel et al. [12] proposed a spatio-temporal action localization approach, they applied a tracking-by-detection model which scored video with a combination of static and motion CNN features. To capture more detail in videos, Krishna et al. [13] utilized contextual information from past and future events to jointly describe all events. Lu et al. [14] proposed a multi-step semantic attention network, which learning visual relation facts as semantic knowledge to help infer the correct answer. However, Video-QA tasks based on vision and natural language require the visual representation of the video combined with subtitles to infer the correct answer, so the Video-QA task is more difficult than image captioning tasks.

The Video-QA task is essentially the fusion of multiple modal data to generate accurate answers to questions related to the video story. Most models for Video-QA, e.g., [15–17] usually use multimodal data to calculate the picture features through the deep convolutional neural network, and the question text features through the recurrent neural network, and then map the input picture and the question features to a common representation space. Finally, the common feature map vector is input to an answer classifier to determine the final answer. However, in real life, the questions people ask about pictures are often related to the target entity in the picture. Therefore, in order to further understand the characteristics of the picture, the image information representation space can be constructed by combining the objects in the picture with the understanding of visual in-formation, and inference the stage focuses on the target entity area of the image and the adjacent caption information. In addition, the characteristics of the questions can be used to focus on different target entity instances according to different questions, thereby improving the accuracy of the answers selected by the model.

In this paper, we propose a novel modal attention fusion model with hybrid multi-head self-attention for Video-QA task based on BERT [18]. We experimented with three Video-QA datasets. First, on the TVQA dataset, we showed that our system outperforms their baseline parser. Second, although the MSVD-QA and MSRVTT-QA datasets are much more challenging and open-ended nature, we are still able to achieve 36.8% and 35.26% accuracy, an absolute improvement of 2.67 points and 0.22 points over baselines, respectively. Additionally, we perform an ablation study and analysis to clarify the strengths of our model, demonstrate the effective-ness of our model in Video-QA. Finally, we demonstrate the effectiveness and advantages of our method over existing methods through question type and required modality experimental results.

## Related work

### Video question answering

A recent direction in Video-QA leverage text modality such as subtitle in addition to video modality for video understanding. It has been gathering a rising attention in recent years, with the release of various Video-QA benchmarks such as STAGE (Lei et al. [19]), it has proposed a Spatio-Temporal Video Question Answering task of requiring intelligent systems to simultaneously retrieve visual concepts of relevant moments to answer spatio-temporal video question, such as a dual-LSTM based approach (Jang et al. [20]) with both spatial and temporal attention, generates spatial and temporal attention to localize which regions in the video need to attend, such as a video question answering framework (Kim et al., [21]) that requires to simultaneously retrieve the relevant moments and referenced visual concepts, such as the progressive attention memory network (PAMN) proposed by Kim et al. [22], the method extracted features through progressive attention mechanism that utilizes cues from both question and answer to progressively prune out irrelevant temporal parts in memory, such as Two-stream method (Lei et al. [23]) based on a bi-directional LSTM to encode both textual and visual sequences. Different from previous studies, we use BERT in our work to model the information captured in the video clips.

### Self-attention for Video-QA

In the past few years, many studies have thus focused on self-attention models, which aim to identify various complex relations to answering questions. For example, Li et al. [24] proposed positional self-attention to simultaneously attend both visual and textual information for improving answer prediction. Kim et al. [25] apply the multi-head attention and self-attention networks to learn the latent concepts in scene frames and captions. Zhang et al. [26] proposed a hierarchical convolutional self-attention encoder-decoder network to efficiently model video contents from videos to answer questions. Jin et al. [27] proposed a multi-interaction network to learn the potential relations between videos and questions. Kim et al. [28] proposed the modality shifting attention network for localizes the temporal moment of interest, and predict the answer using self-attention mechanism on both video and text modality. DFAF framework [29] learn the relationship between multimodalities by adopting the self-attention mechanism.

### Preliminaries

Our work is designed on Video-QA tasks with BERT and Fast R-CNN. In this section, we formally describe the BERT and Faster R-CNN models.

### BERT

We use BERT (Devlin et al., [18]) as the backbone of our model architecture to represent all text data. BERT is a language representation model that used bi-directional Transformers to pre-train on a large dataset, and then used the model parameters of the pre-trained model to fine-tune other NLP tasks. In summary, the BERT model further increased the generalization ability of the word vector model, fully describing the character level, word level and sentence level.

### Faster R-CNN

**Fig 1:**
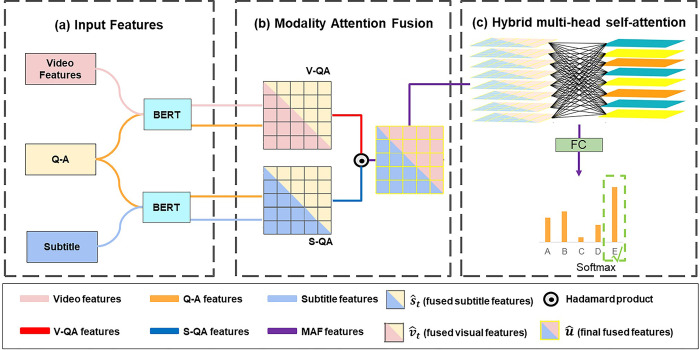
Faster R-CNN network.

We use the Faster R-CNN [30] model to extract visual characteristics. Faster R-CNN is mainly composed of RPN network and VGG16 network. RPN network is used to generate regional candidate frames, and VGG16 network is used to extract feature maps of candidate images. Faster R-CNN detects and recognizes the target in the candidate area based on the candidate frame extracted by RPN. The overall process of Faster R-CNN has 4 units marked with different colors and shown in Fig 1.

1. Feature extraction: Faster R-CNN utilizes the VGG16 network to extract the feature map of the candidate image, which is shared for the subsequent RPN layer and fully connected layer.
2. RPN network: RPN network is used to generate candidate area frames. This layer determines that the anchor point belongs to the foreground or the background, and then uses the bounding box regression to correct the anchor frame to obtain an accurate candidate frame.
3. ROI pooling layer: This layer collects the input feature maps and candidate target regions to extract the feature maps of the target region, and then transmits them to the subsequent fully connected layer.
4. Target classification and regression: Faster R-CNN utilizes the feature map of the target area to calculate the category of the target area, and utilizes the bounding box regression to obtain the final precise position of the detection frame.

## Methodology

**Fig 2:**
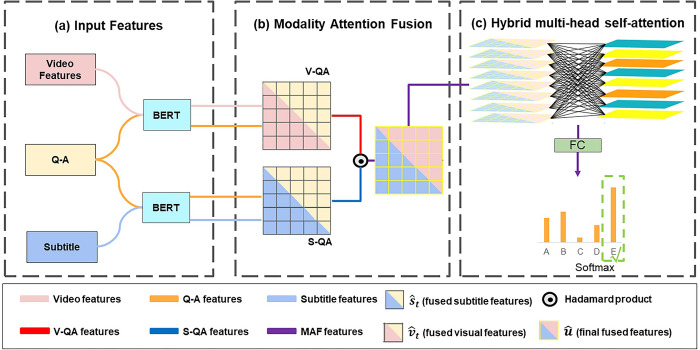
The network architecture of MAF-HMS.

The structure of our proposed framework aims to select the correct answer in the Video-QA tasks (shown in Fig 2). We process video and subtitle features with two independent BERT layers, and use BERT to combine visual conceptual features and subtitles and questions with each candidate answer for embedding. These input features use modal attention fusion (MAF) and a hybrid multi-head self-attention (HMS) mechanism to obtain the final answer prediction.

### Input representation

#### Text representation

We create 5 hypotheses by concatenating a question representation q ∈ ℝ^768^ with 5 candidate answer representations ${\left\{{\text{a}}_{\text{i}}\right\}}_{\text{i}=1}^{5}\in {\mathbb{R}}^{768}$. The question and each of answer candidates were concatenated to form 5 hypotheses $\text{qa}\in {\mathbb{R}}^{{\text{n}}_{\text{qa}}\times 768}$, where n_qa_ represents the maximum number of tokens per hypotheses. For each hypothesis, MAF-HMS learns to predict its correctness score and to maximize the score of the correct answer.

Similarly, we create subtitle representations as $\text{s}\in {\mathbb{R}}^{{\text{n}}_{\text{s}}\times 768}$.

#### Video representation

At first, we extract the sequence of image frames at 3 fps for the video. Then, extract a high-level semantic representation for each image frame. CNN has been recognized as a powerful deep learning model that can capture the visual concept of the image, so our paper uses the Faster R-CNN model to extract visual characteristics $\text{v}\in {\mathbb{R}}^{{\text{n}}_{\text{v}}\times 768}$ from top-20 object proposals. As visual characteristics are in the text domain, they are embedded in the manner as the subtitle.

We extracted video representations $\text{V}\in {\mathbb{R}}^{{\text{n}}_{\text{v}}\times 768}$, text representations $\text{S}\in {\mathbb{R}}^{{\text{n}}_{\text{s}}\times 768}$ in the subtitle, and QA pairs ${\left\{{\text{QA}}_{\text{i}}\right\}}_{\text{i}=1}^{2}\in {\mathbb{R}}^{{\text{n}}_{\text{qa}}\times 768}$ from the second-to-last layer of two BERT layers.

#### BERT

Two separate BERT layers are first applied on the subtitle-QA (s and qa) and Video-QA (v and qa) representations. The pre-trained BERT model can be automatically fine-tuned to achieve the most advanced performance in various NLP tasks. The first token in each input by BERT is [CLS], which is used to obtain the output in the classification task. The [SEP] token is added to indicate the separation between the two inputs. In this article, we consider the tokens of input for two BERT layers as follows:

[CLS]+s+[SEP]+qa

[CLS]+v+[SEP]+qa (1)

The output of BERT layers consists of a set of text and video features. These text and video features are flattened and expressed as $\text{V}\in {\mathbb{R}}^{{\text{n}}_{\text{v}}\times 768}$, $\text{S}\in {\mathbb{R}}^{{\text{n}}_{\text{s}}\times 768}$, ${\left\{{\text{QA}}_{\text{i}}\right\}}_{\text{i}=1}^{2}\in {\mathbb{R}}^{{\text{n}}_{\text{qa}}\times 768}$.

### Modality attention fusion framework

Modality attention fusion framework is designed to fuse video features, subtitle features and QA features into the final MAF features, which are then fed into the hybrid multi-head self-attention layer to obtain the final prediction result.

The QA features ${\text{QA}}_{1}\in {\mathbb{R}}^{{\text{n}}_{\text{qa}}\times 768}$ and video features $\text{V}\in {\mathbb{R}}^{{\text{n}}_{\text{v}}\times 768}$ are fused together as V-QA features ${\text{S}}_{\text{t}}^{\text{V}-\text{QA}}\in {\mathbb{R}}^{{\text{T}}_{\text{QA}}{\times \text{T}}_{\text{V}}}$. Similarly, the QA features ${\text{QA}}_{2}\in {\mathbb{R}}^{{\text{n}}_{\text{qa}}\times 768}$ and subtitle features $\text{S}\in {\mathbb{R}}^{{\text{n}}_{\text{s}}\times 768}$ are fused together as S-QA features ${\text{S}}_{\text{t}}^{\text{S}-\text{QA}}\in {\mathbb{R}}^{{\text{T}}_{\text{QA}}{\times \text{T}}_{\text{S}}}$. Then, we use max pooling operation to reduce the size of the fused features of the two different modalities. The S-QA features ${\text{S}}_{\text{t}}^{\text{S}-\text{QA}}$ as follows:

$$S_{t}^{S-QA}=QA^{T}S_{t}$$ (2)

$$QA_{t}^{S-QA}=S^{T}QA_{t}$$ (3)

$$S_{t}^{att}=softmax(S_{t}^{S-QA})\cdot S_{t}$$ (4)

$$QA^{att}=softmax(QA_{t}^{S-QA^{\text{T}}})\cdot S_{t}$$ (5)

$$S_{t}^{m}=maxpool(fc([S_{t};QA^{att};S_{t}⊙QA^{att}]))$$ (6)

$$QA_{s}^{m}=maxpool(fc([QA;S_{t}^{att};QA⊙S_{t}^{att}]))$$ (7)

Where fc is a fully-connected layer. The fused subtitle features ${\hat{\text{s}}}_{\text{t}}$ from different directions are integrated by concatenating as follows:

$${\hat{s}}_{t}=fc([QA_{s}^{m};S_{t}^{m};QA_{s}^{m}⊙S_{t}^{m};QA_{s}^{m}+S_{t}^{m}])$$ (8)

Similarly, we can define the fused video features as follows:

$${\hat{v}}_{t}=fc([QA_{v}^{m};V_{t}^{m};QA_{v}^{m}⊙V_{t}^{m};QA_{v}^{m}+V_{t}^{m}])$$ (9)

We add fused subtitle features and fused video features to get the final fused features, also called MAF features $\hat{\text{u}}$ as follows:

$$\hat{u}={\hat{s}}_{t}+{\hat{v}}_{t}$$ (10)

### Hybrid multi-head self-attention

More recently, studies on Video-QA task [24–29] show that learning both visual and textual forms of multi-head self-attention leads to more accurate predictions. Multi-head self-attention mechanism is to map the query matrix (Q), key matrix (K) and value matrix (V) to multiple different subspaces. The subspaces are calculated without interference with each other, and finally the output is stitched together. In this paper, $\hat{\text{u}}$ Containing visual and subtitle semantic features are used as input to the multi-head self-attention layer.


$$Q=\hat{u}*W_{i}^{Q},i=1,2,\cdots ,h$$

$$K=\hat{u}*W_{i}^{K},i=1,2,\cdots ,h$$

$$V=\hat{u}*W_{i}^{V},i=1,2,\cdots ,h$$

$$\theta_{s-att}(Q,K,V)=Softmax(\frac{QK^{T}}{\sqrt{d}})V$$ (11)

$$H_{i}=\theta_{s-att}(QW_{i}^{Q},KW_{i}^{K},VW_{i}^{V}),i=1,2,\cdots ,h$$ (12)

$$MulHead=Concat([H_{1};\cdots ;H_{h}])W^{m}$$ (13)

Where ${\text{W}}_{\text{i}}^{\text{Q}}$, ${\text{W}}_{\text{i}}^{\text{K}}$, ${\text{W}}_{\text{i}}^{\text{V}}$ are the linear mapping matrices of the query matrix (Q), key matrix (K), and value matrix (V) in the multi-head attention layer.

However, [31] showed that there is inevitably a limitation called the low-rank bottleneck in the self-attention mechanism. Specifically, increasing the number of heads under the premise of fixed model parameters will lead to a reduction in the size of each head, and a smaller head size will introduce rank constraints on the projection matrices of each head, resulting in a decrease in their expressiveness. Therefore, inspired by [32], we design a hybrid multi-headed self-attentive (HMS) mechanism with the aim of alleviating the low-rank bottleneck issue in multi-headed self-attentive mechanisms for connecting heads to each other to improve the expressive ability of our model.

**Fig 3:**
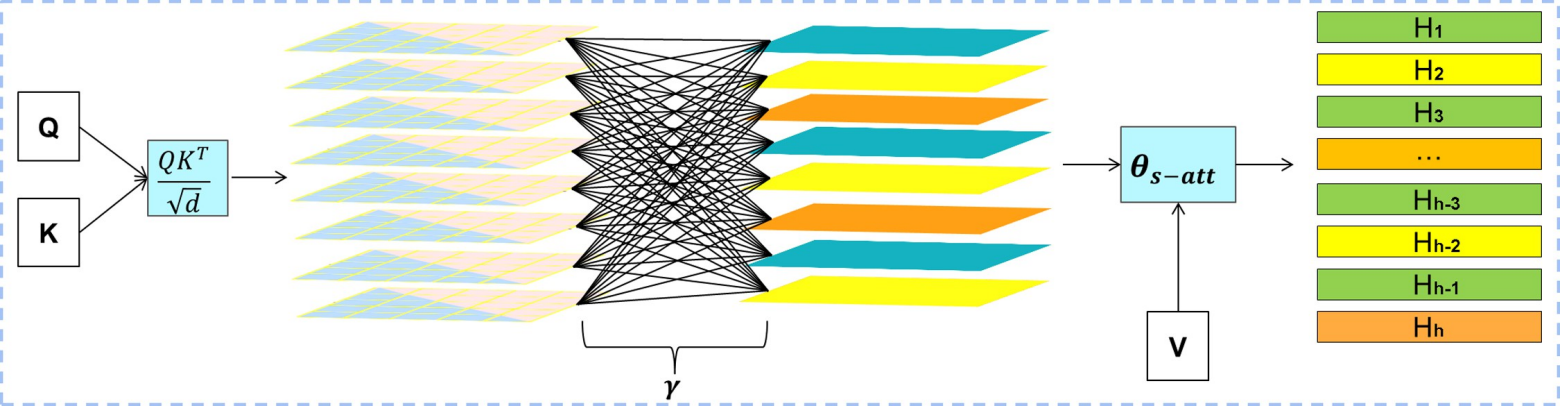
The hybrid multi-head self-attention mechanism.

In the hybrid multi-head self-attention, we add multiple intrinsic inter-vector similarities γ to express complex intrinsic relationships of heads on Eq 11. The hybrid multi-head self-attention model is shown in Fig 3, as illustrated, the model requires only very few additional parameters to allow the model to capture complex multimodal information. $\theta_{\text{s-att}}(\text{Q},\text{K},\text{V})$ is redefined as:

$$\gamma_{i,j}=\left(\begin{array}{ccc} \gamma_{11} & \cdots & \gamma_{1h}\\ \vdots & \ddots & \vdots \\ \gamma_{h1} & \cdots & \gamma_{hh} \end{array}\right)$$ (14)

$$\theta_{s-att}(Q,K,V)=Softmax(\Sigma_{j=1}^{h}\gamma_{i,j}\frac{Q_{i}K_{i}{}^{T}}{\sqrt{d}})V_{i},i=1,2,\cdots ,h$$ (15)

$$H_{i}^{hyb}=\theta_{s-att}(QW_{i}^{Q},KW_{i}^{K},VW_{i}^{V})i=1,2,\cdots ,h$$ (16)

$$\text{Hyb}\_MulHead=Concat([H_{1}^{hyb};\cdots ;H_{h}^{hyb}])W^{hyb}$$ (17)

where h is the number of heads, and we introduce a new matrix $\gamma_{i,j}\in {\mathbb{R}}^{h\times h}$. *γ*_*i*,*j*_ denotes the learnable parameter that can automatically measure the correlation between each head during training.

After obtaining the feature vector through hybrid multi-head self-attention, the probability y of each answer is the correct answer is predicted by the fully-connect and softmax layer:

$$y=Softmax(w_{HMS}*\text{Hyb}\_MulHead(\hat{u})+b_{HMS})$$ (18)

## Experiments

### Dataset

We evaluate our method on three video QA datasets. More details are given below.

#### TVQA [22]

TVQA dataset is a benchmark for Video-QA, containing 152545 human annotated multiple-choice question-answer pairs (84768 what, 13644 how, 17777 where, 15798 why, 17654 who questions), 21.8K video clips from 6 TV shows (The Big Bang Theory, Castle, How I Met Your Mother, Grey’s Anatomy, House M.D., Friends). The questions in TVQA dataset have five answer candidates and only one of them is ground-truth answer. The format of the questions is designed as follows: “[What/How/Where/Why/who]___[when/before/after/…]___?”, and the two part of the question requires visual and linguistic understanding. There are total 122,039 QAs for train set, 15,253 QAs for validation set and 7,623 QAs for test set, respectively.

#### MSVD-QA [33]

MSVD-QA is based on MSVD [34] video dataset. This is a small open-ended dataset of 50,505 question answer pairs annotated from 1,970 short clips. It consists of five types of questions, including what, how, where, when and who, of which 61% of questions are used for training whilst 13% and 26% are used as validation set and test set, respectively.

#### MSRVTT-QA [6]

It is an open-ended dataset that contains 10K videos and 243K question answer pairs. It consists of five types of questions, including what, how, where, when and who. Compared to the other two datasets, videos in MSRVTT-QA contain more complex scenes. They are also much longer, ranging from 10 to 30 seconds long, equivalent to 300 to 900 frames per video. Splits for train, validation and test are with the proportions are 65%, 5%, and 30%, respectively.

### Baselines

We compared our model with several methods:

#### Two-stream (Lei et al., [23])

Combines information from different modalities with LSTMs and cross-attention.

#### PAMN (Kim et al., [22])

Utilize progressive attention memory to update the belief for each answer.

#### Multi-task (Kim et al., [21])

Using Word2Vec and Bi-LSTM for visual and language representations.

#### STAGE (Lei et al., [19])

Using RCNN for visual and BERT for language representations.

### Implementation

In all the experiments, the recommended train/validation/test split was strictly followed, we independently repeated each experiment 10 times and reported average results of ACC. (accuracy).

We use BERT-Base uncased model, it has 12 layers and 768 hidden sizes. In our experiments, the maximum number of tokens per sequence is set to 128, the batch size is 64, the learning rate is set to 0.0001, and the epochs are set to 10. Our evaluation is performed on a machine with Intel(R) Xeon(R) Gold 6132 CPU (2.60GHz), 256G RAM and Nvidia GeForce RTX 2080 Ti.

## Experiment result

### Results

#### Results on TVQA

From **Table 1**, we noted that our approach outperforms the best previous method by 1.53/1.74 accuracy points on the test/val. As compared to the STAGE, we noted the performance is substantially improved. Considering that there are 15,253 and 7,623 validation and test questions, this establishes the strength of our task. We can see that, our model outperforms the baseline models by a large margin. In particular, the scores of our model across all the TV shows are more balanced than the scores from other models, which mean our model is more consistent and robust.

**Table 1:** Evaluation results on the TVQA dataset by TV show.

Model	Accuracy (%)							
	bbt	friends	himym	grey	h.M.D.	cas	all	val.
Two-stream	67.35	62.52	61.78	66.56	62.39	61.46	63.41	63.64
PAMN	67.54	63.47	62.09	67.48	64.17	63.13	64.62	64.65
Multi-task	70.19	65.64	64.02	67.31	66.78	63.87	66.46	66.33
STAGE	71.32	67.03	71.36	70.27	70.56	71.89	70.85	70.47
MAF-HMS	**72.87**	**69.30**	**72.21**	**72.85**	**73.11**	**72.74**	**72.38**	**72.21**

We believe the reasons behind the performance boost are the following:

1. Compared with LSTM-based models (Two-stream, PAMN, Multi-task), BERT-based models (STAGE and MAF-HMS) enable capturing longer dependencies between and within different modalities, especially when there is a long subtitle.
2. Our approach can properly integrate input features from different modalities to help answer questions.
3. Hybrid multi-head self-attention can more fully consider the contribution of each modality, and fusion of multi-head results can make the model extract more important features more accurately and improve the performance of our model.

**Fig 4:**
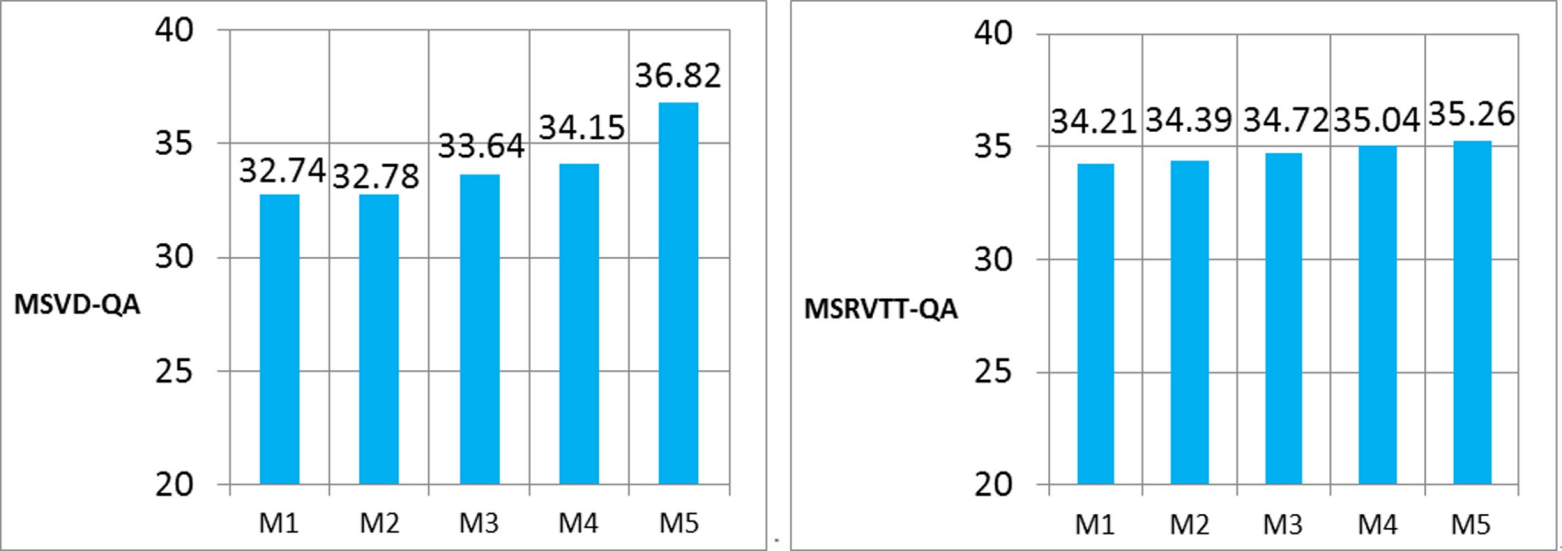
Performance comparison on MSVD-QA and MSRVTT-QA dataset. M1-M5 represent Two-stream, PAMN, Multi-task, STAGE, and MAF-HMS, respectively.

Results on MSVD-QA and MSRVTT-QA. As is shown in **Fig 4**, we compare our MAF-HMS with Two-stream, PAMN, Multi-task and STAGE on MSVD-QA dataset. Our MAF-HMS achieves the most promising performance in overall accuracy, which demonstrates the superiority of the proposed method on the non-trivial scenarios.

The MSVD-QA and MSRVTT-QA datasets represent highly challenging benchmarks for machine compared to the TVQA, thanks to their open-ended nature. Our MAF-HMS model outperforms existing methods on both datasets, achieving 36.82% and 35.26% accuracy which are 2.67 points and 0.22 points improvement on MSVD-QA and MSRVTT-QA, respectively. This suggests that the model can handle both small and large datasets better than existing methods.

### Ablation study

For further analysis, our model shows substantial improvement over the baseline, we evaluated our models with different variants. In these experiments, we choose the same train-and-test setting. From **Table 2**, we have the following observations.

**Table 2:** Ablation study on model variants of MAF-HMS on the validation set of TVQA.

Methods	Model	Val. (%)	Δ (%)
MAF-HMS w/o BERT	GloVe + LSTM	69.57	2.64
MAF-HMS w/o MAF	BERT	68.25	3.96
MAF-HMS w/ VTF (STAGE)	BERT	69.13	3.08
MAF-HMS w/ DMF (PAMN)	BERT	68.52	3.69
MAF-HMS w/ JMCQ (Two-stream)	BERT	68.58	3.63
MAF-HMS w/o HMS	BERT	71.13	1.08
MAF-MS (multi-head self-attention)	BERT	71.76	0.45
MAF-HMS	BERT	72.21	-

#### Effect of model

We design a simpler model with GloVe and LSTM for text representation. As is shown in **Table 2**, we summarize the ablation analysis of MAF-HMS on TVQA dataset in order to measure the validity of the key components of MAF-HMS. Using BERT for contextual word embeddings significantly improves performance comparing to GloVe embedding and LSTM counterpart.

#### Effect of variants

To measure to effectiveness of all components, we evaluate the following settings:

MAF-HMS w/o HMS. Remove the hybrid multi-head self-attention unit in the MAF-HMS.
MAF-MS. To verify the validity of hybrid multi-head attention unit, we employ the standard multi-head attention as a substitute.
MAF-HMS w/o MAF. To measure to effectiveness of MAF component, MAF-HMS w/o MAF underperforms MAF-HMS, which shows that the modality attention fusion framework is important in understanding video questions.
MAF-HMS w/ VTF. We use VTF (Video-Text Fusion, from STAGE) instead of MAF.
MAF-HMS w/ DMF. DMF (Dynamic Modality Fusion) is the modal fusion mechanism of PAMN.
MAF-HMS w/ JMCQ. JMCQ (Video-Text Fusion) is the modal fusion mechanism of Two-stream.

The three “w/” variants have little performance improvement compared to MAF-HMS w/o MAF, and our MAF-HMS has increased by 3.96 points. Thus, MAF component contribution is huge. Finally, we compare our method with two simple variants that drop the HMS and replace the HMS with a standard multi-headed self-attentive unit to show the contribution of our hybrid multi-head self-attention unit. Without HMS, we find out that the performance of our MAF-HMS model decreases by 1.08 points on TVQA dataset. We adopt the multi-headed self-attentive to replace the HMS, the result demonstrates the positive role of HMS.

### Qualitative results

In this section, we provide analysis the qualitative examples of TVQA benchmark solved by MAF-HMS. We selected a few successful and unsuccessful prediction samples from TVQA dataset. Following, we list some of these observations.

For example, the utterance “How did you come to work today” is very important to answer the question, information from neighboring utterances, e.g., “By bus” is the key message to answer the question. All this information is contained in a single modality and is easy to capture, whether it is baselines or our model. Such contextual relationships are prevalent throughout the TVQA dataset. In addition, these are difficult to find the answer information from the subtitles. If there is a lack of key information in the subtitles, such as the question, "what did he hand her", if the context dependence is not obvious in the subtitles, we can find the answer from the video features, e.g., "A man’s hand holding a hamburger". There are strongly related entities in the visual features, our method will give correct predictions based on these fusion features.

We further investigate the performance of MAF-HMS by comparing with STAGE on TVQA dataset. For example, the question “Where did she go after she confessed to him”, there are strongly related entities in the visual features, and the answer can be found from the visual modalities, both MAF-HMS and STAGE obtain accurate prediction for such questions. However, in another question “what did he hand her”, there is some disturbing information with confusion. We can find the wrong answer "diaper bag" from the subtitle "well, this is his diaper bag", and find the correct answer "hamburger" in the video representation, there are strongly related entities in the question features. Other answers can be found in subtitles and video representations, and our model can more accurately eliminate interference options and find the answer directly from the subtitles with understanding the semantics of the question better.

Finally, we show two failure cases. For example, in the question “Who tells Anton that he has quite the temper when interrogating him in the interrogation room?” and the answer is the names of 5 characters, character’s face which is very challenging to capture using the visual concepts extracted by Faster R-CNN. MAF-HMS fails to predict the correct answer as the visual concept features are insufficient in capturing textual cues in the video. Furthermore, in another question “Who is sitting at the computer when the group is talking”, our model is difficult to find the action concept features associated with the question and answer from the subtitles and visual features, so we predict failure. When the answer is not explicit in the video frames or the subtitles, our method gives an incorrect prediction, however our MAF-HMS still achieves outstanding results.

From these examples, we can see that our model is able to solve both visual and language modalities multiple-choice questions.

### Performance by question type

In this section, we evaluate the proposed model with different question types on TVQA dataset.

**Fig 5:**
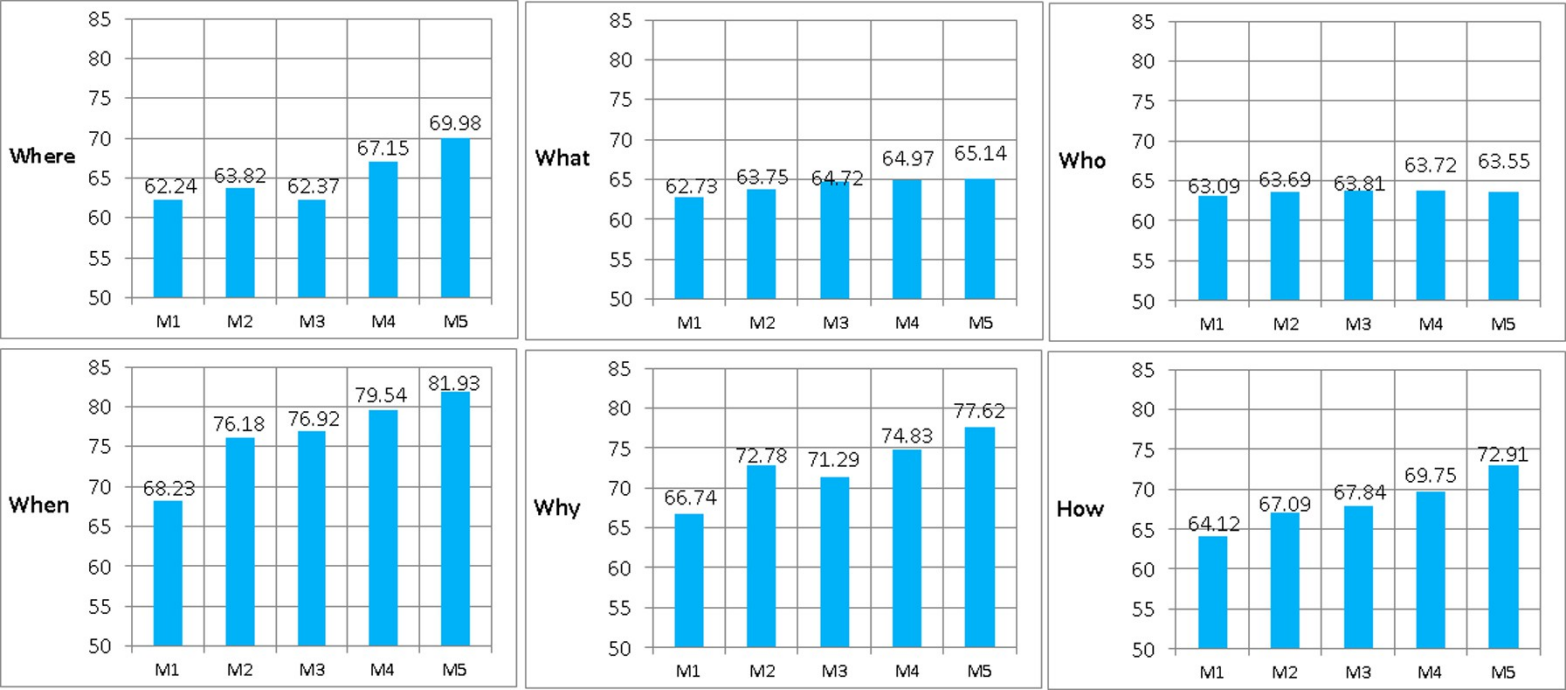
Performance of all the tasks on TVQA dataset by question type. M1-M5 represent Two-stream, PAMN, Multi-task, STAGE, and MAF-HMS, respectively.

This section describes the analysis of MAF-HMS by comparing the accuracy with respect to question type. The **Fig 5** exhibits the performance comparison by 5W1H (Who, Where, When, What, Why and How) question type between Two-stream, PAMN, Multi-task, STAGE and MAF-HMS on the validation set of TVQA benchmark.

For fair comparison with existing methods, we tried to reproduce the results on Two-stream, PAMN, Multi-task and STAGE. Notwithstanding the effect achieved on “who” question is not satisfactory, MAF-HMS obtains an average of 72.19% accuracy for the majority of question types, significantly higher than other baselines. Especially, reached 81.93% accuracy performance on “when” question. The reason is that “when” type sentences contain more interactions and inferences between video representation and text representation, and our model can further integrate patterns with BERT and Faster R-CNN to answer multiple choice questions more effectively. The results confirm that our approach shows additional benefit in mining the modal relations between text and video representation, which implies the superiority of MAF-HMS to help infer the correct answer.

### Performance by required modality

To investigate whether MAF-HMS is sensitive to the modality entities, we provide the analysis of MAF-HMS by the required modality for all datasets. For comparison purpose, we also designed three types of labels according to which modality is required for answering prediction.

**V**. There are only strongly related entities in the visual features.
**S**. There are only strongly related entities in the subtitle features.
**V&S**. There are strongly related entities in the visual features and subtitle features.

**Fig 6:**
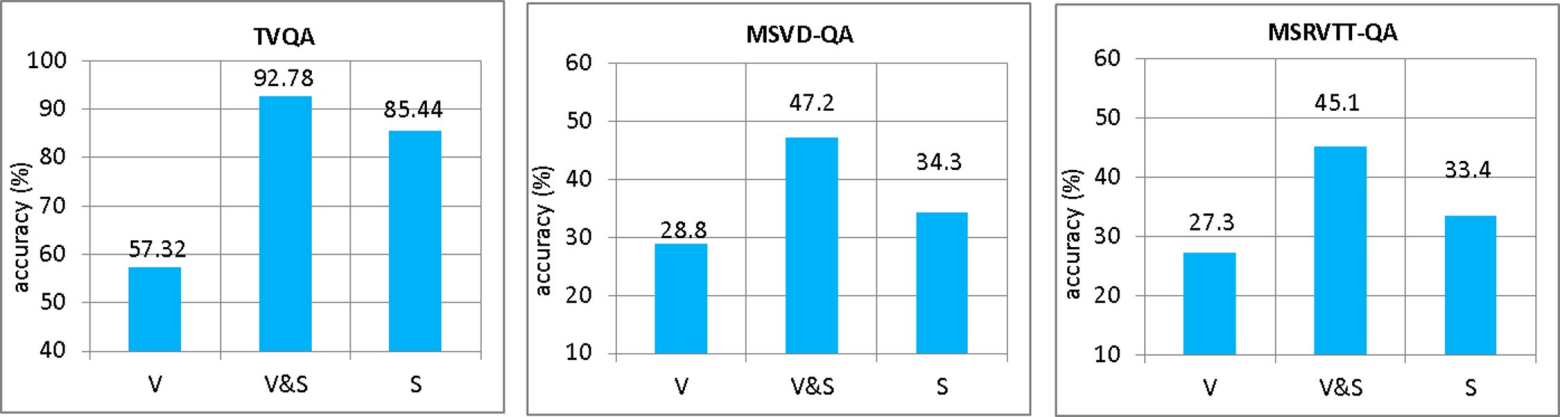
Analysis by required modality of MAF-HMS.

As is shown in **Fig 6**, we have manually labeled 3000 (1000 per each type) examples for each dataset.

It can be seen that require subtitle and video modalities for answer prediction (i.e., label V&S) achieves the highest accuracy score among all datasets, label V has the lowest accuracy score. It means that effectively fusing multi-modal information can strengthen the model’s ability to understand video, and achieving the impressive performance on multi-modal tasks.

## Conclusion

In this work, we presented the MAF-HMS model for Video-QA tasks. We use a modality attention fusion framework that combines visual and subtitles representation features to capture the semantics more accurately. We process video and subtitle features with two independent BERT layers, and use BERT to combine visual conceptual features and subtitles and questions with each candidate answer for embedding. These input features use modal attention fusion and a hybrid multi-head self-attention mechanism to obtain the final answer prediction.

Experiments were conducted to test the performance of our model. Results show that our model gave correct predictions from the language and visual representations on TVQA dataset. In our experiments, the MAF-HMS was evaluated on multiple Video QA datasets, especially, has achieved a test accuracy rate of 72.21% on TVQA dataset. Although the MSVD-QA and MSRVTT-QA datasets are much more challenging and open-ended nature, we are still able to achieve 36.82% and 35.26% accuracy which are 2.67 points and 0.22 points improvement on MSVD-QA and MSRVTT-QA. In addition, we perform an ablation study and analysis to clarify the strengths of our model, demonstrate the effectiveness of our model in Video-QA. Finally, experimental results demonstrate the effectiveness of our method in question type and required modality tasks.

## Supporting information

S1 Dataset (ZIP)
